# Hepatitis B and C Virus Infection and Hepatocellular Carcinoma in China: A Review of Epidemiology and Control Measures

**DOI:** 10.2188/jea.JE20100190

**Published:** 2011-11-05

**Authors:** Masahiro Tanaka, Francisco Katayama, Hideaki Kato, Hideo Tanaka, Jianbing Wang, You Lin Qiao, Manami Inoue

**Affiliations:** 1 Department of Cancer Control and Statistics, Osaka Medical Center for Cancer and Cardiovascular Diseases, Osaka, Japan; 2 Osaka City General Hospital, Osaka, Japan; 3 Department of International Health, University of Tokyo, Tokyo, Japan; 4 Department of Forensic Medicine, Nagoya City University Graduate School of Medical Sciences, Nagoya, Japan; 5 Department of Epidemiology and Prevention, Aichi Cancer Center, Aichi, Japan; 6 Cancer Institute and Hospital, Chinese Academy of Medical Sciences, Beijing, China; 7 Department of Epidemiology, National Cancer Center, Tokyo, Japan

**Keywords:** China, hepatitis, hepatocellular carcinoma, epidemiology, control

## Abstract

China has one of the highest carrier prevalences of hepatitis B virus (HBV) in the world: nearly 10% of the general population. The disease burden of HBV infection and hepatocellular carcinoma (HCC) is also believed to be among the world’s largest, and that of hepatitis C virus (HCV) infection is likely to be substantial as well. However, the epidemiology and measures to control HBV and HCV infection in China remain relatively unknown outside the country. We review the epidemiology of HBV and HCV infection, the disease burden of and risk factors for HCC, and current control measures against HBV and HCV infection in China. We also discuss the relevant literature and implications for future studies of hepatitis and HCC in China.

## 1. INTRODUCTION

Infection with hepatitis B and C virus (HBV and HCV, respectively) and hepatocellular carcinoma (HCC) are responsible for heavy disease burdens in China. In 2006, the Ministry of Health of China (MOH) estimated that, among Chinese aged 1 to 59 years as of 1992, the national prevalence of HBV infection (positivity for HBsAg or any HBV marker) and HBV carriers was 57.63% and 9.75%, respectively, which corresponds to 690 million infected persons and 120 million carriers, as well as 20 million people with chronic hepatitis.^1^ This disease burden is very large, even when compared with that of tuberculosis, which was responsible for 1.4 million new cases in 2000.^2^ Chronic hepatitis B is one of the most serious infectious diseases in China. Unfortunately, we lack a clear picture of the national impact of HCV infection. The nationwide prevalence of HCV infection in 1992 was estimated to be 3.2%, which was higher than in Japan, the United States, and most countries of the European Union. HCC is the second most common malignancy in China, and its most frequent cause is chronic HBV infection.

To date, the MOH has taken several measures to address these diseases. In its National Plan for Prevention and Treatment against Hepatitis B for 2006–10, the MOH states that chronic hepatitis B causes serious consequences for patients, their families, and society as a whole and that it is a major cause of poverty and a health issue of the highest priority.

From a global perspective, China has one of the highest HBV carrier prevalences in the world^3^ and is also estimated to have the highest incidence of HCC (37.9 and 14.2 for males and females, respectively, per 100 000 world standard population as of 2002^4^), accounting for 55% (approximately 340 000 cases) of newly diagnosed cases in the world (approximately 630 000 cases) in 2002.^4^ Despite the magnitude of the burden of chronic viral hepatitis and HCC in China, the epidemiology and control measures for these diseases are relatively unknown outside the country. In this descriptive review, we hope to increase understanding of the trends, the studies that revealed the trends, and the challenges for future research. In selecting studies to be reviewed, we focused on those that were published in internationally accredited English journals and had a full description of their methodology. We also discuss the results of reports in Chinese and Japanese, when information was not available in English.

## 2. PREVALENCE OF HEPATITIS B AND C VIRUS INFECTION

### 2.1 Surveys up to 1990

The first nationwide survey of HBV infection in China was conducted in 1979,^5^ which coincided with the start of economic reform in the country. The overall standardized carrier prevalence was reported to be 8.8%, with a higher prevalence in rural (10.2%) than urban (7.9%) areas. However, this figure may be an underestimate because a reverse passive hemagglutination assay (RPHA) was used for HBsAg testing.^6^ Another survey was conducted in 1980^7^ in 5 provinces; the prevalence of HBV infection was 42.6% and HBsAg carrier prevalence was 10.3% (tested by RIA). The prevalence of HBV infection and HBV carrier status was higher in southern and rural areas than in northern and urban areas. In another survey^8^ from 1984 through 1987 of the 4 provinces of Hunan, Henan, Hebei, and Heilongjiang, the prevalence of HBV infection and HBV carrier status was 58.2% and 10.1%, respectively (tested by RIA).

### 2.2 The nationwide seroepidemiologic survey of hepatitis in 1992

With financial assistance from the World Bank, a nationwide cross-sectional seroepidemiologic survey of hepatitis A, B, C, D, and E infection was carried out in 1992.^9^ It used the Nationwide Disease Surveillance Points (DSPs) system, which was established in 1989. As of 1992, the DSP system comprised 145 reporting sites in 30 province-level divisions (provinces, and provincial-level autonomous regions/municipalities),^10^^,^^11^ which were selected by stratified cluster random sampling. The DSPs covered 1% of representative samples of the Chinese population and have a combined population structure similar to that in the national census. In the survey, 3 subcluster areas, each consisting of about 35 households, were randomly selected from each DSP, and all individuals aged 1 to 59 years in each household were the subjects of the surveillance, for a total of approximately 68 000 subjects. A solid-phase radioimmunoassay was used for detection of HBsAg, anti-HBc, and anti-HBs; EIA was used for HBeAg; and a second-generation UBI EIA was used for anti-HCV. In this section, we summarize and interpret the results of this survey.

#### 2.2.1 Prevalence of hepatitis B infection by age and sex

The estimated overall HBV carrier prevalence among those aged 1 to 59 years was 9.75% (range of prevalence in provinces: 4.49%–17.85%), which was comparable to the results of earlier studies, shown in section 2.1. Age-specific carrier prevalence was 9.67% among those aged 1 to 4 years and 10.22% among those aged 5 to 9 years; it peaked among those aged 10 to 14 years (11.27%). This increasing trend among individuals younger than 15 years suggests the presence of age effects (horizontal transmission) and/or cohort effects. Prevalence was comparable across the ages of 15 to 49 years (range of prevalence in provinces: 9.22%–10.35%). Those aged 50 to 59 had a significantly lower prevalence (7.58%; *P* < 0.01). With regard to sex, males had a significantly higher carrier prevalence (11.3%) than did females (8.2%; *P* < 0.01), as was reported in other countries in East Asia.^12^^,^^13^ The prevalence of anti-HBs, anti-HBc, and HBV infection was 27.42%, 49.81%, and 57.63%, respectively. The age-specific prevalence of HBV infection increased with age from 38.47% among those aged 1 to 4 to 70.69% among those aged 50 to 59. The overall prevalence of HBeAg among HBV carriers was 31.94%, and it decreased with age from 53.32% among those aged 1 to 14 to 12.30% among those aged 40 to 59. Although the prevalence of HBV infection was higher in China, the age and sex characteristics of HBV carriers were comparable to those observed in Japan and Korea.^12^^,^^13^

#### 2.2.2 Prevalence of hepatitis B infection across regions

**Figure 1.**
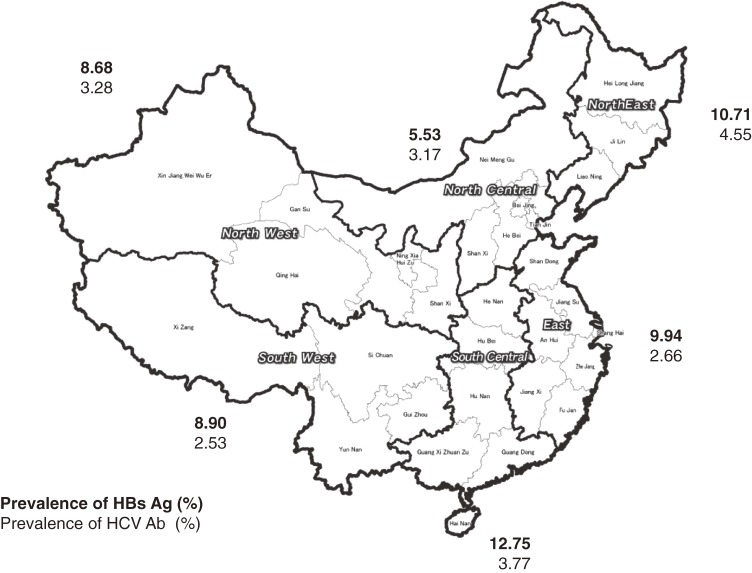
Map of seroepidemiologic survey of hepatitis virus infection in 1992 and prevalence of HBs antigen and anti-HCV by region

HBV carrier prevalence varied across geographic areas. It was significantly higher in rural than in urban areas (10.49% vs. 8.08%; *P* < 0.01). Among the 6 major administrative regions in China as of 1992 (Figure 1), carrier prevalence was 12.75% in the South Central region, 10.71% in the Northeast, 9.94% in the East, 8.90% in the Southwest, 8.68% in the Northwest, and 5.53% in the North Central region. As compared with the national level, carrier prevalence was significantly higher in the South Central region and lower in the Northwest and North Central regions. Geographic disparities in carrier prevalence might represent differences in ethnic distribution, socioeconomic conditions, unsafe medical practices, and access to HBV immunization services.

When the 30 province-level divisions were grouped by HBV carrier prevalence, 10 were classified as high endemic areas (>11%), 11 as moderate endemic areas (7–11%), and the remaining 9 as low endemic areas (<7%). Notably, prevalence was still high even in low endemic areas, as compared with the international standard. In each area, prevalence showed an increasing trend with age up to age 15 years.

#### 2.2.3 Prevalence of HCV infection

The nationwide prevalence of anti-HCV was 3.2% among those aged 1 to 59 years (range of prevalence in provinces: 0.9%–5.1%). It increased significantly with age, from 2.08% among those aged 1 to 4 years to 3.96% among those aged 50 to 59 years. There was no significant difference in prevalence by sex (3.10% for males and 3.30% for females). These prevalences were higher than those reported for first-time Japanese blood donors aged 16 to 64 years in 1995–2000 (0.49%; CI: 0.48%–0.50%)^12^ and the 1988–1994 participants of NHANES III in the United States (1.8%; CI: 1.5%–2.3%).^14^

The difference in the seroprevalence of anti-HCV across administrative regions was less pronounced than that for HBV carrier prevalence. The prevalence of anti-HCV was 4.55% in the Northeast, 3.77% in the South Central region, 3.28% in the Northwest, 3.17% in the North Central region, 2.66% in the East, and 2.53% in the Southwest. As compared with the national level, the prevalence of HCV infection was significantly (*P* < 0.01) higher in the Northeast and South Central regions and lower in the East and Southwest. This regional variation might reflect differences in high-risk practices, such as unsafe medical practices and injecting drug use, as discussed below.

When the 30 province-level divisions were grouped by anti-HCV prevalence, 15 were classified as high endemic areas (3.0%–6.0%) and the remaining 15 as low endemic areas (1.0%–2.9%). In each category, the prevalence of HCV infection tended to increase with age. In the high endemic areas, the prevalence of HCV infection ranged from 2.69% among those aged 5 to 9 years to 5.20% among those aged 40 to 49 years. In the low endemic areas, the prevalence of HCV ranged from 1.37% among those aged 1 to 4 years to 3.07% among those aged 50 to 59 years. The statistically significant rural–urban disparity in the prevalence of HBV infection was not observed in the prevalence of HCV infection (3.42% vs. 3.14%, respectively).

### 2.3 The nationwide seroepidemiologic survey of HBV in 2006

After the introduction in 1988 of a universal HBV vaccination program in China and its gradual expansion (see section 6.1), the MOH conducted a nationwide seroepidemiologic survey in 2006 to assess the prevalence of HBV infection, its risk factors, and the impact of the program.^15^ As in 1992, the 2006 survey used 2-stage clustered sampling. A total of 369 townships were randomly selected (1–4 per county) to represent 160 DSPs in 31 province-level divisions as of 2006 (note: there are 5 levels of local government in China: the province, prefecture/city, county, township, and village). Then, 1 village was randomly selected from each township, and a sample population was randomly selected in each village with a weighted sampling method in the age groups 1 to 4 years, 5 to 14 years, and 15 to 59 years. The total sample population was 82 078. Blood samples were collected, and background information for each person (date of birth, sex, ethnicity, place of birth, occupation, educational level, and immunization history) was also compiled. The immunization status of children younger than 15 years was confirmed by reviewing their immunization record. All serum specimens were tested for HBsAg, anti-HBc, and anti-HBs by ELISA at the National Hepatitis Laboratory in the Chinese Centers for Disease Control and Prevention in Beijing.

The estimated national prevalence among those aged 1 to 59 years was 7.2% for HBsAg, 50.1% for anti-HBs, and 34.1% for anti-HBc. The age-specific carrier prevalence was 1.0% (CI: 0.8%–1.2%) for age 1 to 4 years, 1.4% (CI: 1.2%–1.7%) for 5 to 9 years, 3.2% (CI: 2.6%–3.8%) for 10 to 14 years, 5.4% (CI: 4.4%–6.4%) for 15 to 19 years, and 8.5% to 10.5% for 20 years or older. A marked reduction was observed among those younger than 15 years who participated in the nationwide HBV immunization program, as well as among those aged 15 to 19 years who were partially immunized when the program started in 1988. Using multinomial logistic regression analyses, the risk factors for HBV carrier state among those aged 15 to 59 were identified, namely, lack of HBV immunization (OR: 2.5), male sex (OR: 1.7), and public worker (OR: 3.8) (*P* < 0.01). Among those aged 1 to 14, the major risk factors for HBV carrier state were lack of immunization (OR: 2.5), age 10 to 14 years (OR: 1.9), and birth at a township hospital (OR: 2.1) or at home (OR: 4.0) (*P* < 0.01). Based on these results, the MOH determined that the immunization program contributed to reducing the carrier rate. It also cited the possible beneficial effects of other programs to prevent horizontal infection and promote safe injection.

In addition, the MOH analyzed the immunization status and background information of those born from 1992 to 2005 (age 1–14 years).^16^ Three-dose coverage of HBV vaccination increased from 30.0% for children born in 1992 to 93.4% for those born in 2005. A similar increase was noted—from 22.2% to 82.6%—for those receiving a timely birth dose (ie, within 24 hours after birth). The risk factors for undervaccination (neither a full vaccine series nor a timely birth dose) included older age (5 to 14 years), rural residence, birth at a township hospital or at home, and Tibetan or Uigur ethnicity (*P* < 0.01).

To assess the impact of the HBV immunization program, another survey was conducted in Zhejiang province in 2007^17^ and the results showed a low prevalence (1.52%) of HBV carrier state among those aged 0 to 8 years (*N* = 5047), while the prevalence of anti-HBs was high (65%), which supported the findings of the 2006 survey.

### 2.4 Other reports on the prevalence of HBV and HCV infection

Several other surveys, conducted in limited areas, found prevalences of HBV infection that were comparable with those from the 1992 and 2006 seroepidemiologic surveys, depending on the year they were conducted. Some studies from Tibet^18^ and the Guangxi region^19^ reported very high prevalences of HBV carriers (19%; *N* = 262 and 2132, respectively). With regard to HCV, there are few reports on infection prevalence. The results of those with a comparatively large sample size (*N* ≥ 120) and a detailed description of their testing method^20^^–^^25^ are summarized in Table 1.

**Table 1.** Prevalence of HCV infection in different geographic areas of China

Area (authors)	Reporting year	Characteristics of subjects	No. of subjects	Prevalence of HCV infection	Testing method	Reference no.
Beijing (Sherlock)	1993	apparently healthy people	164	6%	First-generation antibody test	20

Jiangsu (Ito)	1994	blood donors	451	0.7%: anti-HCV 0.2%: HCV RNA	Second-generation antibody test (Dinabo Co, Japan) and ELISA (Ortho Co. USA), and HCV RNA	21

Gansu (Wu, Mizokami)	1995	blood donors (40 volunteer donors and 80 paid donors)	120	Volunteer donor: 2.5%; Paid donor: 35%	EIA 2 (Ortho Diagnostics, Raritan, NJ)	22

Guanxi (Yuan)	1996	hospitalized patients (non-liver disease)	141	0.7%	ELISA Version 2.0 (Ortho Diagnostics, Raritan, NJ)	23

Henan (Zhang, Qiao)	2005	residents aged ≥55 years (participants in an interventional study)	500	9.6%	ELISA Version 3.0 (Ortho Diagnostics, Raritan, NJ)	24

Henan (Liu)	2009	participants in esophageal cancer screening (age 25–65 years)	8226	0.9%	HCV ELISA 3.0 (Autobio Co. Zhengzhou, China)	25

## 3. EPIDEMIOLOGY OF HBV INFECTION

### 3.1 Distribution of HBV genotypes in China

The epidemiologic and clinical characteristics of HBV infection partly depend on virus genotype. In East Asia, genotypes B and C are most prevalent.^26^ Previous studies of genotype distribution in China^27^^,^^28^ showed that genotypes B and C were most common, while genotypes A and D were also present. The distribution of genotypes B and C differed by geographic region. Genotype C was more prevalent in northern provinces, while genotype B was more prevalent in southern provinces. In general, liver cirrhosis and HCC are more likely to result from chronic hepatitis due to HBV genotype C than that due to HBV genotype B. It has been reported that the severity of liver disease induced by HBV genotype B differs by HBV genotype B subgenotype. The Ba subgenotype, which is common in China, has a higher potential for oncogenicity than subtype Bj, which is dominant in Japan.^29^

### 3.2 Carrier status and perinatal and horizontal transmission of HBV

As in other countries in East Asia, perinatal infection plays an important role in the development of HBV carrier state in China. Children born to carrier mothers have a significantly higher risk of becoming an HBV carrier than those born to noncarrier mothers (relative risk 5.3).^30^ Another report found that perinatal infection accounted for 35% to 50% of HBsAg carriers.^31^

Horizontal transmission during childhood is also considered to be common^32^ and an important factor in carrier status. A study in the Guangxi Zhuang Autonomous Region (“Guangxi region” hereafter) showed that, out of 289 children aged 1 to 10 years who had HBsAg-negative parents, 36% were infected with HBV during childhood.^32^ A study from Sichuan province^33^ reported an annual HBV infection rate of 13% among 448 susceptible children in nurseries and preschools.

Previous seroepidemiologic surveys found that horizontal infection had an impact on carrier status. The first nationwide survey, in 1979,^5^ showed increasing carrier prevalence with age: 3.2% among children younger than 1 year, 8.9% among those aged 1 to 4 years, and higher than 10% among those aged 5 to 9 years. A similar prevalence trend was found in the 4-province survey in 1984–87^8^: 3.8% among children younger than 1 year, 8.7% among those aged 1 year, and 9.4% to 12.6% among those aged 2 to 9 years. The results of the nationwide survey in 1992 indicated that when an immunization program was already present in some areas of the country, the impact of horizontal infection was not as clear as in preceding surveys, but increasing trends with age were observed in carrier prevalence, with a peak between age 10 and 19 years. These findings strongly suggest that horizontal infection plays an important role in the development of HBV carrier state in China.

The impact of horizontal infection on carrier status can vary with socioeconomic status and geographic area, with the latter being linked to the genotype distribution of HBV. Some reports estimated that the proportion of individuals with positive carrier status attributable to perinatal transmission was as low as 13% to 20%.^32^ However, it appears that further studies are necessary to confirm these estimates.

### 3.3 Horizontal transmission of HBV among adults

Horizontal transmission has been documented not only among children, but also among adults. The risk of sexual transmission of HBV among newly married couples has also been reported.^34^ In that report, 57 couples comprising 1 HBsAg/anti-HBc positive partner and 1 HBV-susceptible partner (case group) and another 61 couples that were negative for HBV markers (control group) were followed-up for an average of 27 months. HBV transmission was observed in 53% of couples in the case group and only 16% of those in the control group.

Other than sexual and iatrogenic transmission, risk factors for horizontal transmission among adults have seldom been investigated.^35^ However, a case-control study in Shanghai^36^ demonstrated that—in addition to invasive medical procedures, household contact with HBV carriers, and lack of HBV vaccination—body care and beauty treatments were independently associated with the occurrence of acute hepatitis B. That study also found that HBV genotype C2 was an independent risk factor for the progression of acute infection to chronic infection.

### 3.4 Iatrogenic transmission of HBV

Since the 1990s, the World Health Organization has expressed concern regarding the potential transmission of hepatitis viruses due to unsafe medical practices, especially the use of contaminated medical instruments, and has called for implementation of control measures.^37^ Injection is the most common and preferred invasive intervention in medical and preventive (immunization) services in East Asia. As of 2007, China had high prevalences of HBV and HCV infection and an estimated HIV prevalence of 0.1%^38^; thus, reuse of syringes and needles in the absence of sterilization exposes millions of people to the risk of contracting these viruses and other blood-borne diseases.

Because iatrogenic infection can cause substantial health problems, some researchers challenged the necessity and safety of injection services in China.^39^^,^^40^ The national immunization assessment in 2004 showed that only 40% of clinics that provide immunizations used single-use syringes and needles.^41^ Province-level reports are also available. In Shandong province—one of the wealthiest provinces of China—a cross-sectional study of 3 administrative levels of health units (village clinic, township health center, and county hospital) in 1 urban and 1 rural area was performed in 2001. Of 468 health practitioners who provided injection services, 6.2% had unsafe injection practices and 7.6% had improperly handled used disposable syringes.^42^ Another survey of a county in Chongqing city, Sichuan province^43^ found that improperly sterilized glass syringes were found in 52% of health facilities and that injection practices were not correct in 31% of those facilities.

Data on the disease burden from iatrogenic HBV infection in China are limited. A survey by the Centers for Disease Control and Prevention of the Guangxi region showed that 55% of HBV infections in that region were attributable to unsafe injections.^44^ A study in northeastern China, which combined a cross-sectional survey of immunization practice with mathematical modeling of HBV infection, estimated that the annual number of HBV infections due to unsafe immunization injection was at least 135 to 3120 cases among 100 000 fully immunized children.^45^

### 3.5 Other epidemiologic reports on HBV infection

A prospective cohort study of residents in Haimen,^46^ Jiangsu province, a city near Shanghai, found that HBV carriers had a significantly higher risk of dying of non-liver diseases as compared with noncarriers: the relative risk (RR) was 1.2 (CI: 1.1–1.3) in men and 1.4 (CI: 1.1–1.7) in women. When the analysis was limited to all non-liver cancers as the cause of death, the RR was 1.2 (CI: 1.0–1.4) for men and 1.7 (CI: 1.2–2.3) for women. In addition, for non-liver, non-cancer deaths, carriers had significantly higher RRs: 1.2 (CI: 1.1–1.4) and 1.2 (CI: 0.9–1.6) in men and women, respectively. Possible reasons for the increased risk include socioeconomic conditions and behavioral factors associated with carrier status.

## 4. EPIDEMIOLOGY OF HCV INFECTION

In China, reports of epidemiologic studies on hepatitis C are limited, as compared with those on Hepatitis B. As for HBV, the epidemiologic and clinical characteristics of HCV infection partly depend on the genotype of the virus. Several studies reported that the most common genotype identified in China was genotype Ib.^47^^–^^49^

A community-based cross-sectional study of the risk of HCV infection in 4 counties of Hebei province^25^ found that anti-HCV was detected in 0.9% out of 8226 residents aged 25 to 65 years. A subsequent case–control study found that blood transfusion (OR: 4.55), esophageal balloon examination (OR: 3.78), and intravenous injection (OR: 5.83) were significantly (*P* < 0.05) associated with HCV infection.

Some studies of the transmission route for HCV suggested that sexually transmitted diseases and injecting drug use were associated with infection. A follow-up study of heroin users showed an extremely high incidence rate of HCV infection of 37.6 per 100 person-years.^50^ Studies of the prevalence of HCV among injecting drug users showed marked geographic variation; prevalence was 72% in Guangxi region (*N* = 597),^50^ 11% in Shanxi province, and 90% in Hubei^51^ (*N* = 10 724). A meta-analysis based on a systematic review of the prevalence of HCV infection among injecting drug users^52^ reported that the pooled prevalence among injecting drug users in China was 61.4% (CI: 55.7–67.2%) and that the epidemic was most severe in the southern inland provinces of Hubei, Hunan, Yunnan and Guangxi, and the westernmost Xinjiang Autonomous Region. It also found a significant association between infection and ethnic-minority status.

## 5. EPIDEMIOLOGY OF HEPATOCELLULAR CARCINOMA

### 5.1 Mortality and incidence of primary liver cancer (PLC)

Table 2 shows the distribution of mortality rates for the 5 most common anatomical sites for cancer in China during 2004–2005 by sex (data from health statistics of the MOH^53^). Overall, and in males, PLC ranked as the second most frequent cause of cancer death, after lung cancer. In females, it was the third most common cause of cancer death, after lung and stomach cancers. The mortality rate from PLC was higher in males (37.4/100 000) than in females (14.3/100 000). When we compared mortality between urban and rural dwellers (Table 3), HCC was the second most common cause of cancer death in urban areas and the most common cause in rural areas. The slightly lower mortality rate in urban areas (24.4/100 000) than in rural areas (26.9/100 000) might reflect, at least in part, a disparity in carrier prevalence.

**Table 2.** Annual mortality rate (per 100 000 persons) for 5 common malignant neoplasms in China, by sex (2004–5)

Rank	Total		Males		Females	
		
	Disease	Death Rate (%)	Disease	Death Rate (%)	Disease	Death Rate (%)
1	Lung	30.6	Lung	41.1	Lung	19.6
2	Liver	26.1	Liver	37.4	Stomach	16.4
3	Stomach	24.5	Stomach	32.3	Liver	14.3
4	Esophagus	15.0	Esophagus	20.5	Esophagus	9.4
5	Colorectum	7.4	Colorectum	8.3	Colorectum	6.3

**Table 3.** Annual mortality rate (per 100 000 persons) for 5 common malignant neoplasms in China, by area of residence (2004–5)

Rank	Urban		Rural	
	
	Disease	Death Rate (%)	Disease	Death Rate (%)
1	Lung	39.9	Liver	26.9
2	Liver	24.4	Lung	25.7
3	Stomach	22.5	Stomach	25.6
4	Esophagus	10.6	Esophagus	17.3
5	Colorectum	9.7	Colorectum	6.1

Data source: CHINESE HEALTH STATISTICAL DIGEST 2010 by Ministry of Health, China

http://www.moh.gov.cn/publicfiles//business/htmlfiles/zwgkzt/ptjty/digest2010/index.html

**Figure 2.**
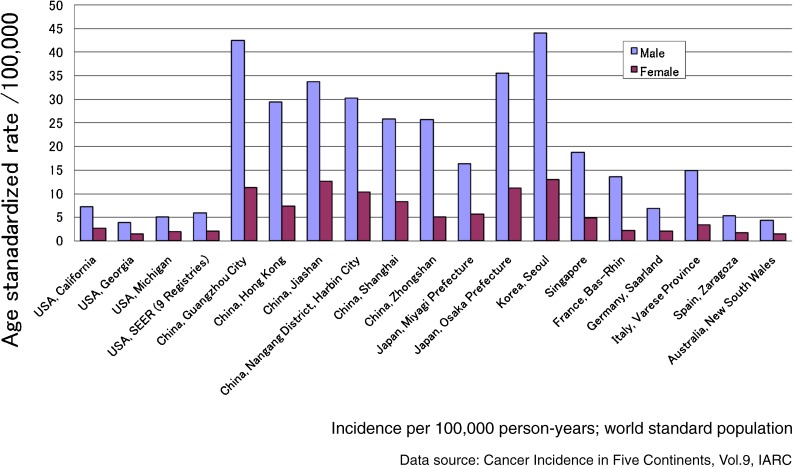
Age-standardized Incidence of primary liver cancer in East Asia, Australia, USA and EU countries, 1998–2002.

Although no incidence data are available for the whole country, Yang et al estimated^54^ the incidence rates for major cancer sites in China in 2005, using mortality rates during 2000–5 and data from 7 cancer registries reported in the Cancer Incidence in Five Continents (CIV) Volume 8.^55^ The estimated incidence rate of HCC was 40.0 in males and 15.3 in females per 100 000 world standard population, ranking it as the second most common cancer, after lung cancer, in both sexes (49.0 in males and 22.9 in females). Using data from CIV Volume 9, Figure 2 shows the annual HCC incidence rate per 100 000 world standard population, as reported in cancer registries in East Asia, Australia, the United States, and selected countries in the European Union. Overall, China has the highest incidence rates.

### 5.2 Temporal trends in PLC incidence

Four Chinese registries that met the data quality requirements of the IARC and appeared in the CIV Volume 9 reported temporal trends in PLC incidence. The age-adjusted incidence rate of PLC has been decreasing in Shanghai city since the 1970s^56^ and in Tianjin city^57^^,^^58^ and Hong Kong^59^ since the 1980s. In Qidong city, near Shanghai, the incidence rate showed no apparent temporal change since 1978, except that the age-specific rate for individuals aged 15 to 34 years has been decreasing during 1988–2002.^60^ In addition to registries in the CIV, there are reports of PLC incidence since the 1970s in Henan, Fujian, and Hebei provinces, but temporal trends varied in these areas.

### 5.3 Risk factors for HCC

#### 5.3.1 HBV infection

There have been several reports on the prevalence of HBsAg among patients with HCC and primary liver cancer.^61^^–^^65^ The results of studies that provided a detailed description of methodology, had a sample size of at least 100, and were published after 1993 are listed in Table 4. Although there were differences in prevalence, due to variation in the methods used for selection of patients and testing for infection, the findings indicated that most HCC cases in China were associated with HBV carrier status. In contrast, HCV was the most important risk factor in Japan, the United States, and the selected European Union countries. Table 5 summarizes recent reports^66^^–^^70^ in English on the RR of HBV carrier state for developing HCC. There are many more Chinese-language reports on relative risk. One systematic review and meta-analysis^70^ of 32, mostly Chinese-language studies, reported a wide range in relative risk (3.8–103). The pooled odds ratio (mOR) was 15.6 (CI: 11.5–21.3) for HBsAg positivity alone and 35.7 (CI: 26.2–48.5) for dual infection with HBV (HBsAg) and HCV (anti-HCV).

**Table 4.** Prevalence of HBV and HCV infection among patients with hepatocellular carcinoma (HCC) in different geographic areas

Area (first author)	Reporting year	Characteristics of subjects	No. of subjects	Virus type	Prevalence of virus (%)	Testing method	Reference no.
Shanghai (Cong et al)	1993	HCC patients	713	HBV	70.8 (Male) 59.7 (Female)	HBsAg	62

Guanxi (Okuno et al)	1994	HCC patients	186	HBV	70	HBsAg	63

10 different regions (Yang et al)	2004	primary liver cancer	3250	HBV	81.0	HBsAg	64

Beijing (Gao et al)	2005	HCC patients	119	HBV	82.4	HBsAg	65

Guanxi (Okuno et al)	1993	HCC patients	186	HCV	5.4	HCV EIA II (Abbott, Dinabot Co, Tokyo)	63

10 regions (Yang et al)	2004	primary liver cancer	3250	HCV	13.2	anti-HCV (details of testing method not shown)	64

Beijing (Gao et al)	2005	HCC patients	119	HCV	11.8	HCV EIA (AxSYM HCV Dynapack Abbott, Tokyo)	65

**Table 5.** Association of hepatocellular carcinoma (HCC) with hepatitis B virus carrier status

Author and year	Reference no.	Study area	Type of study	No. of HCC cases	No. of controls/size of cohort	Person- years observed	RR (95% CI) for HBV carrier state	Adjusted covariates	Other information
Qian et al 1994	66	Shanghai	nested case-control study	55	267	69 393	OR = 7.3 (2.2–24.4)	age, residence, aflatoxin intake assessed by urinary markers	

Zhang et al 1998	67	Henan	case-control study, hospital-based	152	115	—	OR = 28.82 (11.18–78.78)	age, sex	OR = 31.22 (13.86–72.15) (HBV infection)

Yu et al 2002	68	Heimen, Jiangsu	case-control study, population-based	248	248	—	OR = 13.9 (5.78–33.6)	age, sex, residence, history of IV drug use, average income, and eating habits	

Evans et al 2002	69	Heimen, Jiangsu	cohort study, population-based	1092	58 545	434 718	RR = 18.8 (16.0–22.1) (males) RR = 33.2 (17.0–65.0) (females)	age, sex, history of acute hepatitis, family history of HCC, occupation, current alcohol and tea intake, history of drinking well water	end-point was HCC death

Shi et al 2005	70	reports from China	meta-analysis based on systematic review	3201	4005	—	mOR: 15.6 (CI: 11.5–21.3)	—	based on 32 case-control studies reported from 1966–2004, mOR is for HBsAg-positive and HCV-Ab-negative state

Note: Hepatitis B virus infection status was identified by the presence of HBs antigen; only English-language reports that were published after 1990 and adjusted for potential confounders were selected.

Abbreviations: CI: confidence interval, HBV: hepatitis B virus, HCV: hepatitis C virus, RR: relative risk of developing HCC, OR: adjusted odds ratio by multiple logistic regression analysis, mOR: odds ratio obtained by meta-analysis, HCC: hepatocellular carcinoma.

#### 5.3.2 HCV infection

Since the 1990s, there have been several reports on the prevalence of HCV infection among HCC or primary liver cancer patients. As mentioned above, the reported prevalences are summarized in Table 4. Prevalence can partly depend on patient selection and the testing method, but these findings suggest that the prevalence of HCV infection among patients with HCC varies by geographic area.

Regarding the relative risk of HCV infection for developing HCC, there are few reports in English that include a detailed description of the study methodology. Among them, reports from Haimen city^68^ and Henan province^67^ reported aORs of 0.77 (CI: 0.19–3.18) and 2.57 (CI: 0.57–12.03). The abovementioned systematic review of studies in Chinese^70^ reported an mOR of 8.1 (CI: 5.0–13.0) for anti-HCV-only positivity.

#### 5.3.3 Alcohol intake

Worldwide, alcohol intake is considered a probable cause of HCC.^71^ Analytical studies in China that assessed risk, after adjusting for HBV carrier status, have had variable results. A case-control study from Henan province^72^ found that an alcohol intake of 50 g/day or more at least once a week for 1 or more years was associated with a weak but significant risk (aOR: 1.06; *P* < 0.05). They also found a dose-response relationship between alcohol intake and development of HCC, namely, heavy drinkers (>5000 grams of alcohol per month) had an approximately 3- to 4-fold risk as compared with nondrinkers. This increased risk was not significant in another case-control study from Haimen city^68^ that compared ever- and never-drinkers (aOR: 1.38, CI: 0.68–2.81). A prospective cohort study from Haimen city found no increased risk associated with drinking 4 or more drinks/week (aRR: 0.57 for males and 0.9 for females). Another cohort study that observed 145 male HBV carriers for 10 years also found no association between HCC incidence and consumption of more than 1000 mL/month of alcohol.^73^ The differences in the findings of these studies might be partially due to variation in the criteria for and confirmation of alcohol consumption and/or adjustment for confounding factors such as HCV infection.

#### 5.3.4 Smoking

China has a very high prevalence of smoking among males, and the disease burden from tobacco is believed to be substantial. One study estimated that 1 in 4 smokers was killed by smoking in the 1980s.^74^ The relationship between smoking and the development of HCC in China has only recently been studied. A case-control study in Haimen city that estimated the relative risk of smoking after adjusting for HBV carrier state and alcohol intake,^68^ and a cohort study of HCV carriers in the same area, found no increased risk due to smoking.^73^ In contrast, a cohort study in Shanghai that followed-up 18 244 men^75^ found that the risk of dying from HCC was significantly higher (aRR: 1.8; *P* < 0.05) for smokers who smoked 20 or more cigarettes per day as compared with never smokers, after adjusting for alcohol use. More recently, a large-scale case-control study was carried out to determine whether smoking was a cofactor for HCC development.^76^ It analyzed data from 36 000 people who died of HCC as cases and 17 000 who died of liver cirrhosis as controls from 24 cities and 74 rural counties, to represent geographic differences. The RR for liver cancer death, standardized for age and locality, showed a 36% excess risk of death among male smokers aged 35 years or older (RR: 1.36; CI: 1.29–1.43; 2*P* < 0.000 01; attributable fraction, 18%) and 17% excess risk among female smokers (RR: 1.17; CI: 1.06–1.29; 2*P* = 0.003; attributable fraction, 3%). These figures suggest that approximately 50 000 liver cancer deaths are caused by smoking every year in China. Another, more recent report showed that the attributable fraction of liver cancer due to smoking was 18.72% for men and 0.95% for women.^77^

#### 5.3.5 Aflatoxin

Aflatoxins are classified as established carcinogens by the IARC^78^ and are considered an important cause of HCC in some developing areas in the world. In China, exposure to aflatoxins is the second most-studied risk factor for HCC, after hepatitis viruses. It is hypothesized that the decreasing incidence of HCC in Hong Kong and Singapore is at least partially due to the decrease in aflatoxin contamination in food, which resulted from economic development.^79^

Regarding the relationship between aflatoxin and HCC, several ecological studies^80^^–^^82^ were reported in the 1980s, but the results were not consistent. Later, some case-control studies^68^^,^^72^ found a significant correlation between HCC and intake of peanuts and corn, which are believed to be among the most contaminated foods in China. In these studies, exposure to aflatoxin was assessed by estimates of aflatoxin ingestion obtained via population-based estimates of food intake, food sampling analyses, or in-person food frequency interview. Because these indirect estimates of exposure were not sufficiently reliable, a more recent nested case-control study in Shanghai^66^ used urinary aflatoxins and their metabolites as a marker for exposure. It showed that the RRs for HCC development among noncarriers exposed to aflatoxin, unexposed HBV carriers, and exposed carriers were 3.4 (CI: 1.1–10.0), 7.3 (CI: 2.2–24.4), and 59.4 (CI: 16.6–212.0), respectively, when unexposed noncarriers were used as the reference. The results suggested that aflatoxins were risk factors for both carriers and noncarriers and that positive interaction existed between carrier status and aflatoxin exposure. These results were consistent with those of 2 independent cohort studies of HBV carriers in Qidong City, which showed that the RR of aflatoxin exposures for development of HCC, as assessed by the presence of urinary aflatoxin markers and the 249ser-p53 mutation, was 3.3 (CI: 1.2–8.7; attributable risk: 0.553)^73^ and 3.5 (CI: 1.5–8.1),^83^ respectively.

#### 5.3.6 Other risk factors

Familial clustering of HCC has been documented.^84^^,^^85^ A population-based case-control study in Taixing city, Jiangsu province^86^ demonstrated that relatives of HCC patients had a significantly increased risk (OR: 3.06; CI: 1.48–6.33) of HCC as compared with relatives of controls, after adjusting for age and sex. However, after adjustment for HBV carrier status, the OR was no longer statistically significant. On the other hand, a case-control study in Henan province^72^ and a cohort study in Jiangsu province^69^ found that a familial HCC history was a risk factor, with aORs of 11.80 (CI: 2.75–50.61) and 2.3 (CI: 1.9–2.7; males only), respectively, independent of HBV carrier status, alcohol intake, or exposure to crops potentially contaminated with aflatoxin. However, these studies could not identify the specific causes for the increased risk associated with kinship.

Some studies have suggested that drinking contaminated pond water is a risk factor for development of HCC.^87^ This potential risk has been attributed to microcystin produced by algae. However, this association was not confirmed by other reports,^68^ and it is possible that this potential risk factor was confounded with other risk factors.

The Yangtze valley is known for a high endemicity of schistosomiasis, which causes liver cirrhosis. A case-control study in Sichuan on the development of HCC and its association with a previous diagnosis of schistosomiasis^88^ found that infection was significantly associated with HCC (OR: 3.7; CI: 1.0–13.0). However, research in Japanese provinces where the infection was endemic found that the association with HCC was caused by a confounder, ie, hepatitis virus infection.^89^ The association between schistosomiasis infection and HCC development is currently considered less likely because the infection does not cause major inflammation in the hepatic parenchyma.

As described in section 5.1, the incidence and mortality rate of HCC are higher in males than in females in China. This difference can partly be explained by sex differences in the carrier prevalence of HBV. In addition, a cohort study in Haimen city^90^ found a significant synergistic effect in the interaction between sex and HBV infection on liver cancer mortality, after adjusting for smoking and alcohol intake. That study also found that approximately 60% of male deaths from HCC might be attributable to this synergistic effect alone.

## 6. CONTROL MEASURES AGAINST HEPATITIS B

### 6.1 HBV immunization

The first hepatitis B vaccine was derived from human carrier plasma and was licensed in the United States in 1981. As described above, China has had a very large disease burden from chronic HBV infection, and the introduction of HB vaccination was an urgent priority. However, there were challenges in procuring and financing the huge number of vaccines needed for an annual birth cohort of more than 20 million in the 1980s. To overcome these challenges, local production of the vaccine was mandated. In 1986, plasma-derived vaccines were locally produced for the first time in China, and clinical trials confirmed them to be safe and highly effective in preventing HBV infection in infants born to HBV carrier mothers.^91^ In 1988, the vaccine was introduced to some areas of the country.^31^ Because the supply was limited, the vaccine was mainly used for neonates born to HBV carrier mothers. As a result, the prevalence of carriers in Beijing among children aged 0 to 7 years decreased from between 5% and 7% to between 3% and 4%.^31^

To overcome the vaccine shortage and potential risk of plasma-derived vaccine, the MOH started a project to produce the vaccine from a recombinant expression system. In 1996, a Chinese hamster ovary (CHO)-cell HBsAg expression system was licensed to produce recombinant CHO hepatitis B vaccine. At approximately the same time, production of a yeast-based recombinant vaccine was started after a technology transfer from Merck & Dohme Co. The recombinant vaccine was confirmed to be safe and effective in clinical trials and eventually replaced the plasma-derived vaccine. Since 2001, all hepatitis B vaccine used in China has been recombinant (yeast or CHO).^91^

In 1992, the MOH started universal vaccination for newborns. This led to increased coverage in urban and areas of high socioeconomic status, but the coverage was less extensive in rural and less affluent areas. A national review of immunization in 1999 showed that immunization coverage with 3-dose HB vaccine was 70.7% for the whole country, but it varied from 99% in Beijing to 7.8% in Tibet. The lower coverage in less affluent areas was due to the cost of vaccination paid by parents, a lack of public awareness of the vaccine, and inadequate vaccine supplies.^91^

To raise coverage, the MOH began to provide all neonates with free vaccine in 2002 under the National Plan for HBV Immunization,^92^ but parents were still charged an injection fee (US$1.10).^15^ Meanwhile, together with the Global Alliance for Vaccines and Immunization, the MOH initiated a project to provide free vaccination to all neonates in 12 western provinces and some less privileged areas in other provinces. A survey in 2005 of 11 of the western provinces showed that average timely birth-dose coverage increased to 88% among neonates born in township hospitals.^91^ After this success in the western provinces, the Chinese government introduced a policy in 2005 to provide all vaccines listed in the National Immunization Program free-of-charge to all neonates and infants in the country. Thereafter, it was reported that HB vaccine coverage in neonates was maintained at nearly 95.0% in urban areas of the country and that coverage in most rural areas increased to between 83.5% and 96.5%.^91^

The current HB vaccination schedule in China is based on that of the Expanded Program on Immunization of the WHO.^93^ The first dose is given within 24 hours of birth, and the second and third doses are given in the second and sixth months. This schedule is based on a report^94^ indicating that approximately 90% of vertical transmission can be prevented without using HB immunoglobulin if the first dose is given within 24 hours of birth.

### 6.2 National Plan for Prevention and Treatment of Hepatitis B, 2006–10

In early 2006, the MOH adopted the National Plan for Prevention and Treatment against Hepatitis B (NPHB) for 2006–10. The NPHB was a reflection of the Ministry’s acknowledgment that hepatitis B was a critical health challenge affecting patients, their families, and society as a whole, and that control of the disease was still insufficient despite the success of the immunization program. The NPHB had 3 major objectives, namely, (1) to reduce carrier prevalence to lower than 1% among children aged 0 to 5 years, (2) to reduce carrier prevalence in the overall population to lower than 7%, and (3) to reduce carrier prevalence by 1% in provinces where carrier prevalence among all residents was already lower than 7%. To achieve these objectives, the NPHB established a surveillance and testing system for HBV, strengthened the immunization program, enhanced safe injection practice in medical and immunization services, promoted knowledge of hepatitis among general public, and trained health care workers. Progress in implementation of the NPHB was assessed annually and its activities were modified accordingly.

While vertical transmission of HBV was controlled by the HBV immunization program, the NPHB also proposed measures to control horizontal infection. Among these measures was prevention at home, including HBsAg testing before marriage and immunization for noncarrier spouses, promotion of condom use for carrier spouses, and immunization of all noncarrier family members in a household with a carrier.

As mentioned above, prevention of iatrogenic infection is very important in heath care settings; however, reports on the implementation of safe injection measures are limited. A 6-year interventional study in village clinics in Hebei province reported that, after training health practitioners in clinics and providing single-use injection materials, HBV carrier prevalence among 2-year-old children born to noncarrier mothers dropped to 2.1%; prevalence was 11.6% in a control group with no intervention.^32^

In heath care facilities, provision of safe blood is also important. We discuss this topic in the next section.

### 6.3 The blood banking system and promotion of safe blood provision

Because of the high prevalence of HBV and HCV and the increasing numbers of HIV carriers,^95^ provision of safe blood is a critical issue in China. The Chinese Society of Blood Transfusion reported that 3.1% and 1.1% of all whole blood donors in China were seropositive for HBsAg and anti-HCV, respectively, in 1999.^96^ We briefly describe the blood banking system, based on the limited literature written in English and Japanese by Chinese authors.

Until the 1970s, provision of blood in health care settings depended on paid donors recruited by each facility. During the Cultural Revolution, the availability of blood and its safety were further compromised due to social instability. To ensure a safe blood supply, a compulsory blood donation system for citizens was introduced in 1978, and a national standard for blood donor qualification and donation practices was implemented by the MOH for the first time.^97^ The system, however, retained a partial payment system, due to the presence of a donation quota for each employer and an honorarium for donation. The acceleration of economic reforms in the 1990s was accompanied by an increase in the number of unlicensed private blood collection centers, some of which used unsafe blood collection methods. For example, non-sterilized needles were often reused and donors did not undergo HIV testing. Donor-to-donor transmission of blood-borne viruses also occurred in these centers due to pooling of blood from multiple plasma donors and returning of red cell components to each donor.^96^ Among current HIV carriers in China (approximately 1.5 million in 2010),^95^ approximately 10% of HIV infection is attributable to these unsafe blood collection and provision practices.^98^

To ensure a stable blood supply and safety in blood transfusion, the government passed the Blood Donation Law in 1998.^99^ The provisions of the law include strengthening of a nonremunerated blood donation system, clarification of the responsibility of local governments to secure and promote safety in blood donation, establishment of blood donation and supply centers at each level of local government, and establishment of systems and regulations in blood donation/provision and clinical practice, such as donor qualification, a blood screening system, and standards for blood transfusion practice at health care facilities.

Based on the provisions of the law, the blood donation and banking system came under the authority of the health department of the central and local governments. A provincial central blood center, a city central blood station, and prefectural blood donation stations were established. As of 2000, 95% of the 3 levels of local governments for the whole country were reported to have established blood centers/stations, including 325 central blood stations at city level or higher.^97^ The requirements for donors were defined as a healthy person aged 18 to 55 years weighing 50 kg or more for men or 45 kg or more for women. The minimum interval between donations was 6 months. Donated blood was required to be screened for hemoglobin concentration and packed-cell volume, ABO group, HBsAg, anti-HCV, ALT, anti-HIV1/2, and syphilis infection. With the introduction of the Blood Donation Law, the proportion of blood donated by volunteers for clinical use increased from 11% in 1996 to 67% in 2000.^96^ This proportion is believed to be increasing in urban areas. In Shanghai, for example, 90% of all donors in 2007 were volunteers.^100^ Also, a compensation system for patients infected with HCV through blood transfusion was established there in 1996.^101^ According to a recent report, the volume of blood used for transfusion in China continues to increase as a result of the increasing amount of surgery, and blood shortages occur even in Beijing during winter and summer, when students, the major donor population, temporarily leave the capital.^98^

## 7. DISCUSSION

China has the largest population and one of the largest territories in the world. Its diversity in socioeconomic conditions, ethnicity, and culture has a considerable influence on disease characteristics. The incidence and mortality of hepatitis and HCC, and their epidemiologic patterns, vary across the country. Therefore, it is not possible to fully describe the characteristics of these diseases with data from only a few areas, and we must be cautious in interpreting these reports. The scientific literature on hepatitis and HCC in China has been increasing very rapidly in the past decades, but we need more time before a clearer national picture of the epidemiology of these conditions emerges. In addition, English-language reports and assessments of recently introduced projects or systems, such as the HBV vaccination program and the establishment of a blood banking system, are still scarce. Thus, we also need to accumulate more balanced information on these developments.

The national seroepidemiologic survey in 1992 provided valuable information on the prevalence and epidemiology of hepatitis virus infection in China. The report, however, had some limitations, including an inadequate description of its methodology, a lack of references to validate information sources, and the absence of confidence intervals for prevalence data. Moreover, the survey’s finding of an apparent limited impact of the HBV vaccination program among cohorts born after 1988 casts doubt on the appropriateness of the sampling and testing methods. Nevertheless, the results were consistent with observations in other studies: (1) as described in section 2, HBV carrier studies in various parts of the country showed prevalences ranging from 5% to 20%, which are consistent with values from subgroup analyses in the national survey, (2) a study of carrier prevalence among 22 707 Taiwanese born in various provinces of the mainland^102^ showed that the overall carrier prevalence of HBV was 15.2% (testing by RIA), with variation by native province and a higher prevalence among those born in southern provinces. Therefore, the survey results are probably sufficiently reliable in describing the basic epidemiologic trends in hepatitis virus infection in China as of the early 1990s.

The prevalence of HCV infection varied considerably across reports from different areas in China. Some areas showed that the prevalence of HCV infection was higher than that in Japan, the United States, and European Union countries, in which the average prevalence is typically below 3%.^103^ This variability might represent not only real differences in prevalence, but also differences in sampling and testing methods. However, the main sources of HCV infection in China are believed to be iatrogenic transmission and injecting drug use, both of which are associated with transmission risks that vary greatly according to the distribution of unsafe medical practices and high-risk populations in the society. Thus, it is also likely that there is wide variability in true carrier prevalence across geographic areas and population subgroups.

A systematic review of the RR of HBV carrier state for the development of HCC in China showed a wide range of ORs (3.8–103.0). During the 1980s and 1990s, studies in Japan showed a similarly wide range (6.9–58.2).^104^^–^^107^ These results are roughly comparable, but any assessment of risk in these 2 countries must take into account the differences in methodology, particularly in the sampling method and adjustment for confounding factors such as alcohol use, smoking, and HCV carrier status. The pooled OR of HCV carrier state for the development of HCC in China was somewhat lower (mOR: 8.1) than ORs reported in Japan (range: 9–101).^108^^–^^111^ This divergence might have been caused by differences in average life span, distribution of HCV genotypes, and prevalences of other risk factors, including alcohol consumption, which is higher per person in Japan than in China.^112^ Additional data and analyses are needed in both China and Japan to identify the causes for this difference.

In China, HBV is the most important risk factor for the development of HCC. Although the HBV immunization program is expected to greatly reduce HCC incidence, it will require a few more decades before we start to see an obvious decrease among the general population. With the high cost of the current antiviral therapy for chronic HB, HCC control among existing carriers depends on the reduction of risk factors that accelerate the development of HCC among carriers. Among the risk factors we reviewed, only smoking, aflatoxin exposure, and alcohol intake are preventable by intervention measures. However, not all HCC patients were exposed to these risk factors, and the importance of obesity, diabetes, and other potential risk factors for HCC among carriers has yet to be carefully assessed. We need additional evidence on risk factors in order to develop more effective plans for their reduction.

China is a very important field for studying the relationship between aflatoxin and HCC development, due to the endemicity of this environmental carcinogen and HBV. Studies cited in this review appear to have established a causal link between aflatoxin exposure and HCC, at least among HBV carriers. However, there is still limited evidence from human biological monitoring on the geographic distribution of aflatoxin contamination, although contamination in agricultural crops is relatively well documented. Further studies are needed to assess the population attributable risk of aflatoxin contamination on the disease burden of HCC in China.

In addition to host (human) and environmental factors, viral characteristics are also important in the development of HCC. Among these characteristics, information on HBV genotype has rapidly accumulated in the past decades.^113^^,^^114^ Data on genotype, however, are still difficult to obtain in population-based epidemiologic studies. Researchers need to consider the potential effects of genotype when interpreting the results of epidemiologic studies.

With the introduction of HBV immunization, control of HBV infection is substantially progressing in China, which will lead to a dramatic decrease in the disease burden from HBV infection and HCC. However, the national prevalence of HCV is not negligible, and some areas have a very high prevalence. The comparative disease burden from HCV infection is expected to increase as the proportion of HBV-immunized cohorts grows in the country. There is as yet no promising candidate for a vaccine against HCV, and the current standard treatment, interferon, is not affordable for the general population in China. Under these conditions, the only obvious measure to curb the disease burden from HCV infection is prevention of its transmission, particularly through the iatrogenic route. To clarify the epidemiology and disease characteristics of HCV infection and related HCC, further studies are needed. It might therefore be beneficial for China to consider cooperation with other countries that have been actively studying these diseases.
